# Comparison of Different Approaches for Testing Sorption by a Superabsorbent Polymer to Be Used in Cement-Based Materials

**DOI:** 10.3390/ma13215015

**Published:** 2020-11-06

**Authors:** Peihua Zhong, Jun Wang, Xiaoxian Wang, Jiaping Liu, Zhen Li, Yichuan Zhou

**Affiliations:** 1 School of Materials Science and Engineering, Southeast University, Nanjing 211189, China; 230159484@seu.edu.cn (P.Z.); 230189198@seu.edu.cn (J.W.); 230198209@seu.edu.cn (X.W.); 2 Jiangsu Sobute New Materials Co., Ltd, Nanjing 211103, China; lizhen@cnjsjk.cn; 3 State Key Laboratory of High Performance Civil Engineering Materials, Nanjing 211103, China; 4 School of Materials Science and Engineering, Chongqing University, Chongqing 400045, China; 20152833@cqu.edu.cn

**Keywords:** superabsorbent polymer, absorptivity, absorption method

## Abstract

The absorption and desorption behavior of superabsorbent polymer (SAP) can influence various properties of cementitious materials. Therefore, it is essential to know these performances of SAP prior to implementation in cement-based materials. In this paper, two types of SAP with different chemical compositions were tested in free liquid (deionized water and cement filtrate) and cement paste. Five absorption test methods were considered, including the tea-bag method, the filtration method, the centrifuge method, the suction filtration method, and the slump flow method. The results show that the absorptivity of SAP A73 and SAP N in cement paste by the slump flow method are about 21 g/g and 7 g/g, respectively. In addition, the centrifuge method and suction filtration method give more accurate absorption values when compared to the tea-bag method and filtration method due to their effectiveness in removing inter-particle liquid. Though the absorptivity obtained by the tea-bag method is higher than the centrifuge method and suction filtration method, it is still a good pre-test method to reveal the performance of SAP used in cementitious materials due to time-saving and simple setups.

## 1. Introduction

Superabsorbent polymer (SAP) are some kind of polymer hydrogels. Normally, SAP are covalently crosslinked with polyelectrolyte chains to form a three-dimensional polymer network [1]. In the past few years, as a kind of chemical admixtures for cementitious materials, SAP were widely investigated because of their multiple functionalities. SAP can be used for mitigation of autogenous shrinkage [2,3,4], rheology modification [5,6,7], improvement of freeze-thaw resistance [8,9], mitigation of chloride penetration [10,11], reduction of fire spalling [12,13], and self-healing [14,15,16]. In all these potential applications, the absorption and desorption kinetics of SAP are important factors affecting the performance and microstructure of cementitious materials [17,18,19]. Frequently, SAP will be added to the concrete mixture in a dry state during mixing. As a result, SAP will absorb water rapidly during mixing. Then water-filled inclusions are formed once contacting with mixing water. This swelling capacity of SAP depends both on the nature of the SAP and the composition of the mixture [20,21,22]. When the SAP reached their final absorption capacity, stable sizes of water-filled inclusions were formed. Then, the water present in the inclusions will redistribute into smaller capillary pores due to capillary pressure and later participate in the hydration reaction [23]. After the pore solution in the inclusions is consumed, the inclusions end up as empty macro pores remain in the concrete matrix [23,24]. Thus, measuring and predicting the absorption and desorption behavior of SAP would be necessary for its applications into cementitious materials. Incorrect estimation of the absorption and desorption behavior of SAP may have a negative impact on the properties of cement-based materials [25,26].

Currently, both the tea-bag method and filtration method are widely used for measuring the absorption behavior of SAP, which are recommended by RILEM (International Union of Laboratories and Experts in Construction Materials, Systems and Structures) for testing sorption by SAP before implementation in cementitious materials [27]. For the tea-bag method, the inter-particle liquid is removed by wiping with a dry cloth. The treatment for removing the inter-particle liquid by the filtration method is to suspend the funnel with filter paper and wait until all the excess liquid is drained out by gravity. However, it has been postulated by several researchers that there is potential inaccuracy resulting from the residual inter-particle liquid remaining in wet SAP samples during the testing procedure of both methods [20,22,28]. During both the tea-bag method and filtration method, SAP is exposed to a free liquid environment. In cementitious systems, SAP is surrounded by aggregates, cement particles, or other solid components. Therefore, SAP will compete for the limited mixing water with cement particles in cementitious systems. In addition, testing liquid (e.g., synthetic pore solution, cement slurry filtrate, or saline solution) may be different with the real pore solution in cementitious materials due to the ionic composition of pore solution in cementitious materials may change over time [29]. When the slump flow method was used in real mortar, the lower absorptivity of SAP was observed when compared with the tea-bag method [30]. However, the effectiveness of removing inter-particle liquid by the tea-bag method has not yet been verified. For the filtration method, the treatment of removing inter-particle liquid seems to be unsuitable for cement-based materials. During the process of filtration, SAP samples are immersed in excess test liquid. SAP may continue to absorb the solution or may release the solution already absorbed [20,22]. Furthermore, the absorption capacity of SAP will decrease when they are used under an external load [31,32]. Thus, the absorption under load (AUL) of SAP is a good method to reflect the swelling properties of SAP under pressure (e.g., applications in personal care products and agriculture) [33].

Several other methods were applied in a real cementitious environment for the test of absorptivity. Mönnig [34] compared the slump flow as a function of the mixing time to predict the absorptivity of SAP. According to Sun et al. [35], the extra water content for SAP can be estimated by the flowability of mortar.

However, this method can only estimate the absorption of SAP in cementitious materials during the early age due to the limitation of mixing and setting time. Johansen et al. [36] developed a method to determine the absorptivity of internal curing materials. The early age heat evolution data obtained by isothermal calorimetry was used to estimate the absorptivity. However, this approach can be used only if the water absorbed by internal curing materials will not participate in the early hydration of cement. This is inconsistent with the evidence that cement paste with internal curing have a higher degree of hydration compared with the reference cement paste when the total water to cement ratio (w/c) are the same [37,38]. Based on the Delesse’s principle [39], the area fraction of the SAP pores obtained by a plane section can represent the volumetric fraction of these pores in the specimen. Zhao et al. measured the absorptivity of SAP by an optical microscope based on the area fraction of SAP voids in polished cross sections of cement paste and concrete. Just et al. [40] employed SEM images to study the absorption capacity of a solution-polymerized SAP. It should be noted that the polished cross sections of the specimen may not intersect all the centers of SAP voids. Therefore, the diameters of SAP voids obtained by cross sections are smaller than the real situation. In recent years, some advanced instruments and technology (i.e., neutron tomography [41], neutron radiography [1,7], and nuclear magnetic resonance [42]) have been used to examine the absorption and desorption kinetics of SAP in cement-based materials. Though these advanced technologies can give more accurate results, these methods not only require specialized experimental equipment but are also time consuming. In addition, to evaluate whether a kind of SAP is suitable for using in cementitious materials, the real cementitious materials test is not necessary. Therefore, the pre-test to evaluate the suitability of SAP need to be easy to operate and are practical.

The aim of this paper is to verify the applicability and variability of five simple methods, including the tea-bag method, the filtration method, the centrifuge method, the suction filtration method, and the slump flow method. The tea-bag method and filtration method are followed by the recommendation of RILEM TC 260-RSC 27. Considering the inaccuracy caused by inter-particle liquid for both the tea-bag method and filtration method, the modified tea-bag method and filtration method were also considered based on previous studies [20,28,43].

The modified tea-bag method (centrifuge method) can remove the inter-particle liquid by centrifugal force after taking the tea-bag out of the test liquid. The modified filtration method (suction filtration method) can remove the inter-particle liquid by vacuum suction pressure right after planned times. As for the absorptivity of SAP in cement-based materials, it is estimated by the slump flow method. By comparing the absorptivity of SAP obtained in free solution and cement paste, the effectiveness of these simple methods to predict the absorptivity in cementitious materials can be evaluated.

## 2. Materials and Methods

### 2.1. Materials

**Figure 1:**
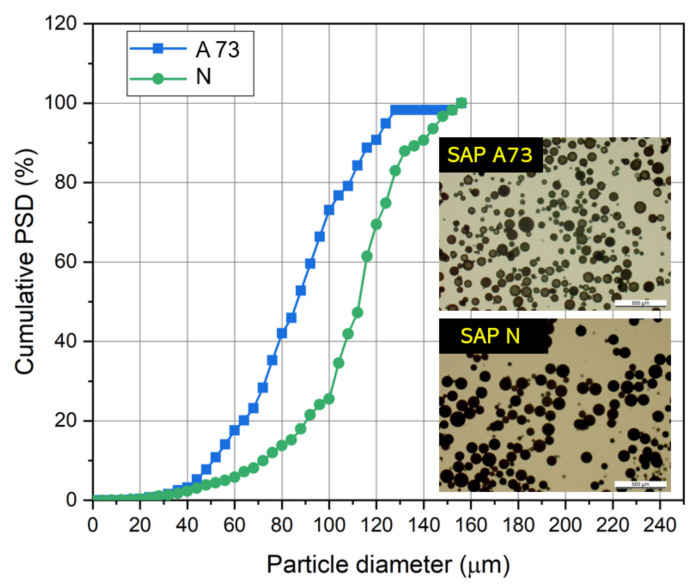
Shape and particle size distribution of used superabsorbent polymer (SAP).

Two SAP types were used, SAP N and SAP A73, which were both synthesized by means of inverse suspension polymerization and in the form of spherical particles. SAP N was produced of acrylamide as the sole main monomer. SAP A73 is a crosslinked copolymer composed of the two main monomers acrylic acid (partially neutralized with NaOH) and acrylamide. Both SAP were crosslinked with N-N’-methylenebisacrylamide (MBAM) and the cross-linking density was similar. Both SAP have already been examined in our previous work [38]. The general recipe and specific synthesis process are also shown in Reference [38]. The SAP density of SAP N and SAP A73 are 1.1 g/mL and 1.5 g/mL, respectively. In Figure 1, the particle shapes and cumulative particle size distributions (PSD) for both SAP types are presented (determined by optical microscope image analysis).

Ordinary Portland cement (CEM I 42.5) from China United Cement Co., Ltd (Beijing, China) was used to prepare cement filtrate and cement paste. The phase composition (in wt.%) of cement is calculated according to the Bogue method [44] as C2S (31.5), C3S (40.7), C3A (2.1), and C4AF (10.5). The density and Blaine fineness of cement are 3.11 g/mL and 356 m2/kg, respectively. A commercial polycarboxylate-base superplasticizer (PCE, 40% solid contents) used in this study is provided by Jiangsu Sobute New Materials Co., Ltd (Nanjing, China).

As the absorption capacity of SAP depends on the test liquid, both deionized water and cement filtrate were applied. Cement and deionized water with w/c = 5 was used to prepare the cement filtrate, as recommended by 27. After 24 h of automatic stirring, the cement filtrate is filtered to obtain cement filtrate (pH = 12.93). All measurements (including the absorption test) were run in the same climate room at 25 ± 1 °C and 70 ± 3% RH.

### 2.2. Tea-Bag Method

At first, 10 individual dry tea-bags were used to examine the average mass ($m_{0}$) of the test fluid absorbed by a dry tea-bag. The mass of a dry tea-bag was weighed ($m_{1}$), approximately 0.05 g. Then this dry tea-bag was filled with a dry SAP sample and weighed ($m_{2}$). The dry tea-bag filled with dry SAP was put into a plastic bottle, which contains test solution and is sealed with a cap to reduce carbonation and evaporation. After 1, 5, 10, 30, 60 min, 3, and 24 h, the wet tea-bag with swollen SAP was taken out from the plastic bottle. Before weighing, this tea-bag was placed on a dry cloth and gently wiped with another one for around 15 s to remove the inter-particle liquid. Then, the mass of the wet tea-bag with swollen SAP was weighed ($m_{3}$). This wet tea-bag with swollen SAP was put back into the plastic bottles and sealed with a cap again until the next measuring. Each SAP sample were measured by three individual tea-bags. According to Equation (1), the absorption capacity (AC) of SAP can be calculated. The amount of SAP should be adjusted according to the actual absorption of SAP from the pre-test. If the amount of SAP is too high, the swollen SAP may exceed the volume of the tea-bag and escape from the tea-bag into the test solution. At the same time, the tea-bag may also hinder the free sorption behavior of SAP 27.

(1) $$\mathrm{AC}\;=\;\frac{m_{3}-m_{2}-m_{0}}{m_{2}-m_{1}}$$

### 2.3. Filtration Method

At first, an amount of 0.05 g of dry SAP (exact mass $m_{1}$) was placed in an empty plastic bottle. Then, the plastic bottle was filled with an excess of test liquid (approximately 100 g, exact mass $m_{2}$) and sealed with a cap to reduce carbonation and evaporation. After the scheduled time (5 min, 10 min, 30 min, 60 min, 3 h, and 24 h), the whole solution was filtered by a suspended funnel with pre-wetted filter paper. Then, the mass of filtered fluid was weighed ($m_{3}$). Three individual measurements were performed for each SAP sample. The absorption capacity (AC) of SAP can be obtained by Equation (2). The SAP particles are still immersed into the test liquid during the filtration time (approximately 5–10 min). Thus, the absorption capacity at 1 min cannot be measured. The total contact time with the test solution is dependent on the speed of filtration [45]. In order to avoid the influence of setups, the type of filter paper and the funnel was kept the same in each measurement.

(2) $$\mathrm{AC}\;=\;\frac{m_{2}-m_{3}}{m_{1}}$$

### 2.4. Centrifuge Method

**Figure 2:**
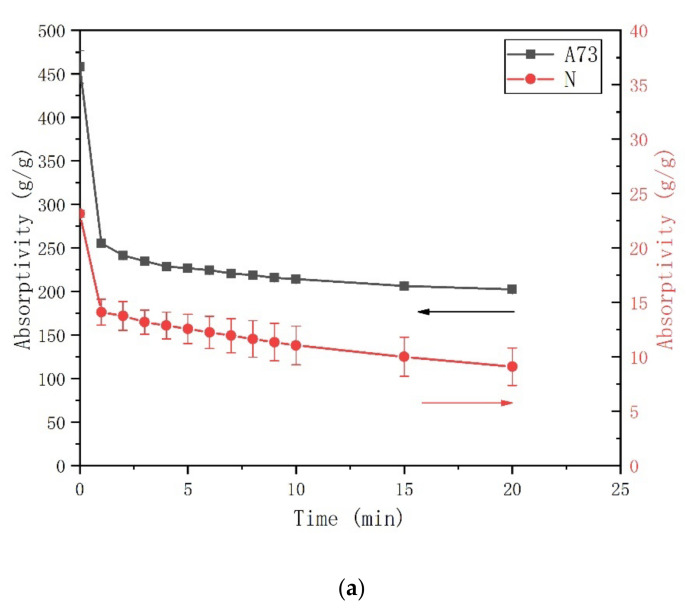

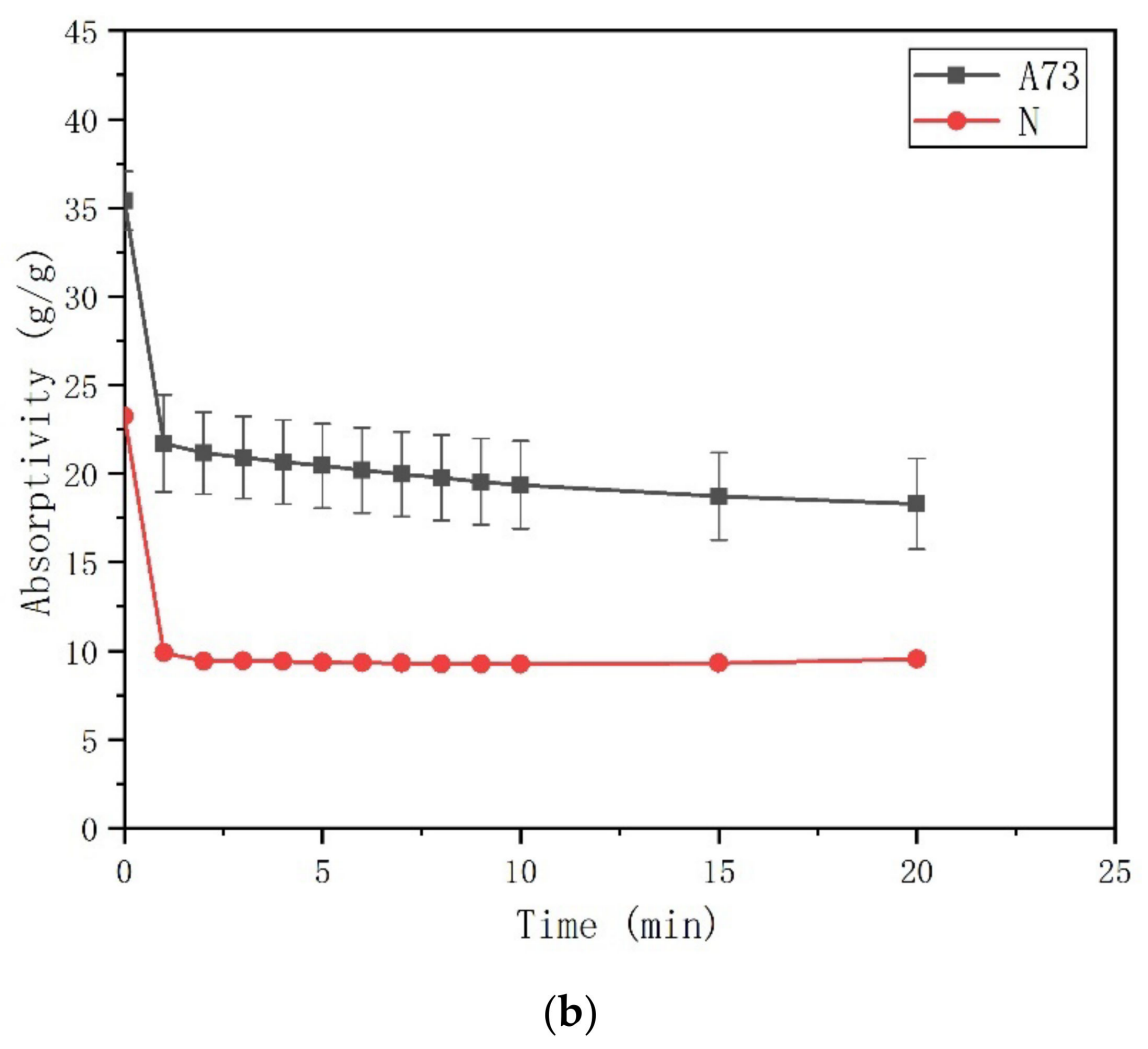
Absorption of the superabsorbent polymer (SAP) at 30 min as a function of centrifugation time: (**a**) deionized water and (**b**) cement filtrate.

Similar to the tea-bag method, the mass of a dry tea-bag was weighed ($m_{1}$) at first. Then, approximately 0.05 g of the dry SAP was added into this dry tea-bag and weighed ($m_{2}$). This tea-bag was put into a plastic bottle which contained excess test solution and was sealed with a cap to reduce carbonation and evaporation. After a predetermined time (1 min, 5 min, 10 min, 30 min, 60 min, 3 h, and 24 h), the tea-bag was removed from the plastic bottle and placed in a centrifuge tube with a filter sieve. When the centrifugal speed reaches 1000 rpm, the excess liquid can be removed by 20. The mass of the tea-bag with swollen SAP after centrifugation was weighed ($m_{3}$). With this method, 10 individual tea-bags were used to examine the average mass ($m_{0}$) of fluid absorbed by a dry tea-bag. Three individual tea-bags were measured for each SAP sample. According to Equation (3), the absorption capacity of SAP can be estimated. The accuracy of this method is affected by the centrifuge time. Thus, different centrifuge times were examined to estimate the duration of centrifugation (see Figure 2). In all measurements, the absorptivity of SAP decreases with increasing centrifugation time. The absorptivity decreases fastest in the first minute. Then the decrease rate remained nearly constant. This indicates that the excess liquid, including the inter-particle liquid, was removed during the first minute. Thus, the duration of centrifugation is set as 1 min.

(3) $$\mathrm{AC}\;=\;\frac{m_{3}-m_{2}-m_{0}}{m_{2}-m_{1}}$$

### 2.5. Suction Filtration Method

An amount of 0.05 g of dry SAP (exact mass $m_{1}$) was placed in an empty plastic bottle. This plastic bottle was then filled with excess test liquid and covered with a cap to reduce carbonation and evaporation.

After a predetermined time (1 min, 5 min, 10 min, 30 min, 60 min, 3 h, and 24 h), the whole solution with SAP samples was poured into a Büchner funnel and filtered with suction pressure. To ensure all the wet SAP particles were poured into the Büchner funnel with test liquid, the plastic bottle was washed with test liquid and the wash liquid was also poured into the Büchner funnel. The filter paper was fluid saturated by a test liquid prior to filtration. The suction pressure produced by a vacuum pump was kept around 0.03 MPa [28].

The process of filtration should be stopped when there is no drop below the funnel. The mass ($m_{2}$) of the pre-wet Büchner funnel and filter paper was measured after the first filtration. The amount of Büchner funnel with a wet SAP sample after the second filtration was weighed in determining the mass ($m_{3}$). All measurements were performed in triplicate. The absorption capacity (AC) of SAP was given by Equation (4).

(4) $$\mathrm{AC}\;=\;\frac{m_{3}-m_{2}}{m_{1}}$$

### 2.6. Slump Flow Method

According to Mönnig [46] and Sun et al. [35], the absorptivity of SAP in cement pastes can be estimated by comparing the slump flow of mixtures containing SAP with reference mixtures. The w/c of reference cement pastes ranged from 0.3 to 0.4 and the interval is set to 0.02. The mixture proportions of cement pastes are shown in Table 1. If the slump flow of a mixture with SAP is similar with that of a reference mixture, the available water in both mixtures should be the same. Accordingly, the absorption capacity (AC) can be determined by Equation (5). To obtain a proper flowability and avoid the influence caused by a different amount of superplasticizer, the amount of superplasticizer was kept constant between the different mixtures (0.1% by mass of cement). Mostly, the dosage of SAP used in cementitious materials for internal curing is 0.3–6% by the weight of cement [30]. In this study, the amount of SAP added in cement pastes was kept 0.3% by weight of cement. Each paste was mixed with the same mixing process. Dry SAP and cement were mixed for 1 min at first. Then, the mixing water and pre-dissolved superplasticizer were added and wet mixed for 5 min. After mixing, the slump flow can be measured by following the Chinese National Standard GB/T 2077–2000 [47].

(5) $$\mathrm{AC}\;=\;\frac{m_{added}^{water}-m_{available}^{water}}{m_{polymer}}$$

where $m_{available}^{water}$ represents the mass of water in the reference mixture, $m_{added}^{water}$ is the mass of water in the SAP mixture, which has a similar slump flow to the reference mixture, and $m_{polymer}$ is the mass of SAP in the mixture.

## 3. Results and Discussion

### 3.1. Absorption of SAP in Deionized Water

**Figure 3:**
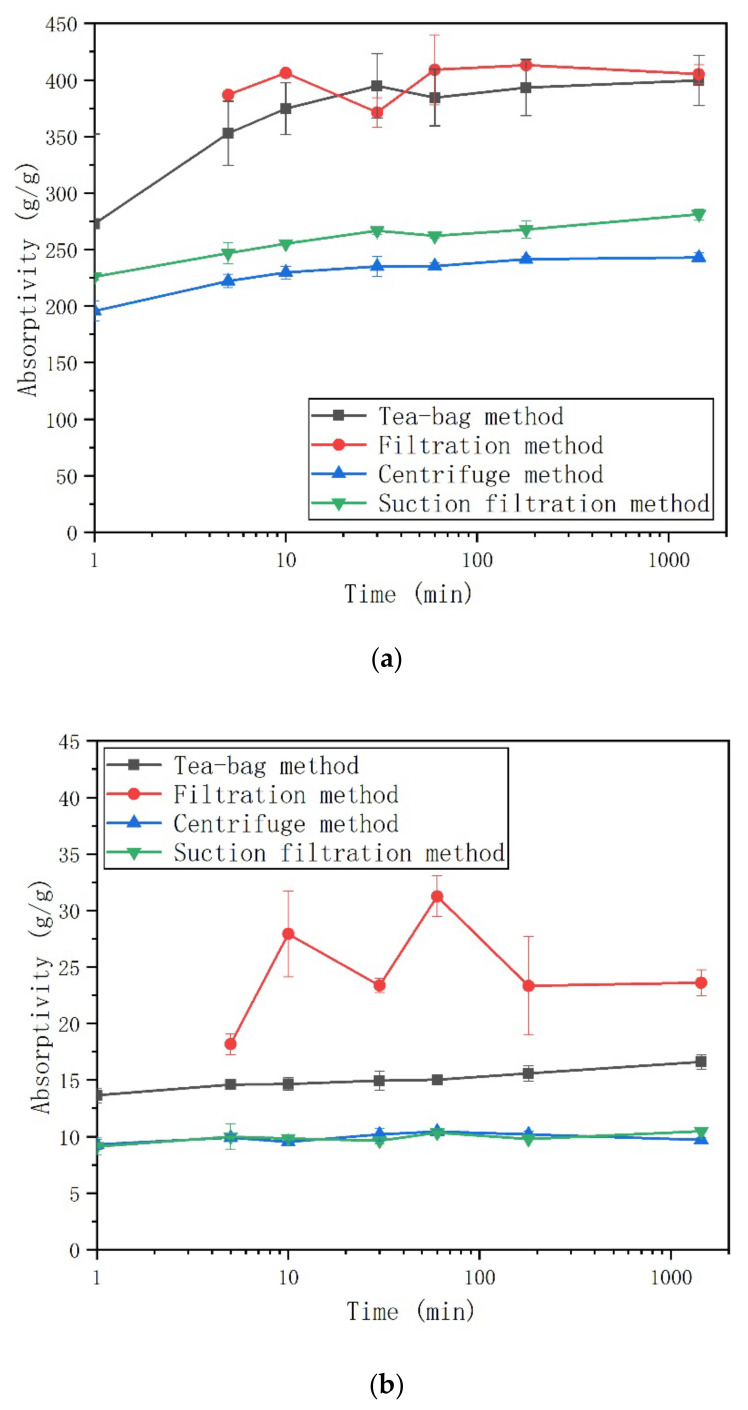
Development of SAP absorption in deionized water measured with different methods: (**a**) anionic SAP A73 and (**b**) AM-based SAP N.

In Figure 3, the absorption kinetics of both SAP in deionized water measured by different methods are presented. In deionized water, the absorption kinetics of both SAP observed by different methods are very similar. After a rapid initial intake of water at a short period of time, both SAP reached their saturated absorptivity. The maximum absorptivity of SAP A73 is much higher than that of SAP N in deionized water. The reason comes from the different driving force for the expansion of the different SAP types [21]. According to Flory’s theory [48], the absorptivity of SAP is attributed to the crosslinking density, ionic osmotic, and affinity of the polymer toward fluid. Since the crosslinking agent contents for both SAP were kept the same [38], the absorptivity was dependent on the ionic osmotic and/or affinity of the polymer. For SAP A73, osmotic pressure is the main driving force for swelling [49]. Nevertheless, the water affinity plays the main role for the swelling behavior of SAP N, which comes from the hydrophilic amine group on the network [50]. In addition, the driving force comes from the water affinity being weaker when compared with the osmotic pressure. Accordingly, SAP A73 shows higher absorptivity in deionized water.

When compared to the centrifuge method and suction filtration method, the scatter of the tea-bag method and filtration method were higher for both SAP types. For SAP A73, the scatter for the tea-bag method and filtration method ranges from 22 g/g to 32 g/g and the scatter for centrifuge method and suction filtration method ranges from 2 g/g to 9 g/g. For SAP N, the scatter for the tea-bag method and filtration method ranges from 0.2 g/g to 5 g/g. The scatter for the centrifuge method and suction filtration method ranges from 0.1 g/g to 1.2 g/g. Most likely this is due to the different methods and SAP types applied. The higher scatter will increase the risk of inappropriate mixture compositions when SAP is added in cementitious materials. The amount of SAP used in cement-based materials usually depend on the absorption behavior of SAP characterized by a particular method. However, the results here show the absorptivity of SAP in deionized water, which is different from the real situation of the cement environment (see Section 3.2 and Section 3.3).

The first measurement time of filtration method is at 5 min because the filtering process of 100 g deionized water without SAP is already beyond 1 min. When compared with the other three methods, a higher absorptivity of SAP was observed during the early age (e.g., before 10 min) and less time has been taken to reach its maximum absorptivity. This difference is caused by the process of filtering [22]. In other words, since SAP is still exposed in the test solution during the filtering process, the absorptivity measured at a testing time may, in reality, be a later value. At a later stage, the delay of weighing due to filtering time have little influence on the absorptivity because SAP already reached its maximum absorptivity.

**Figure 4:**
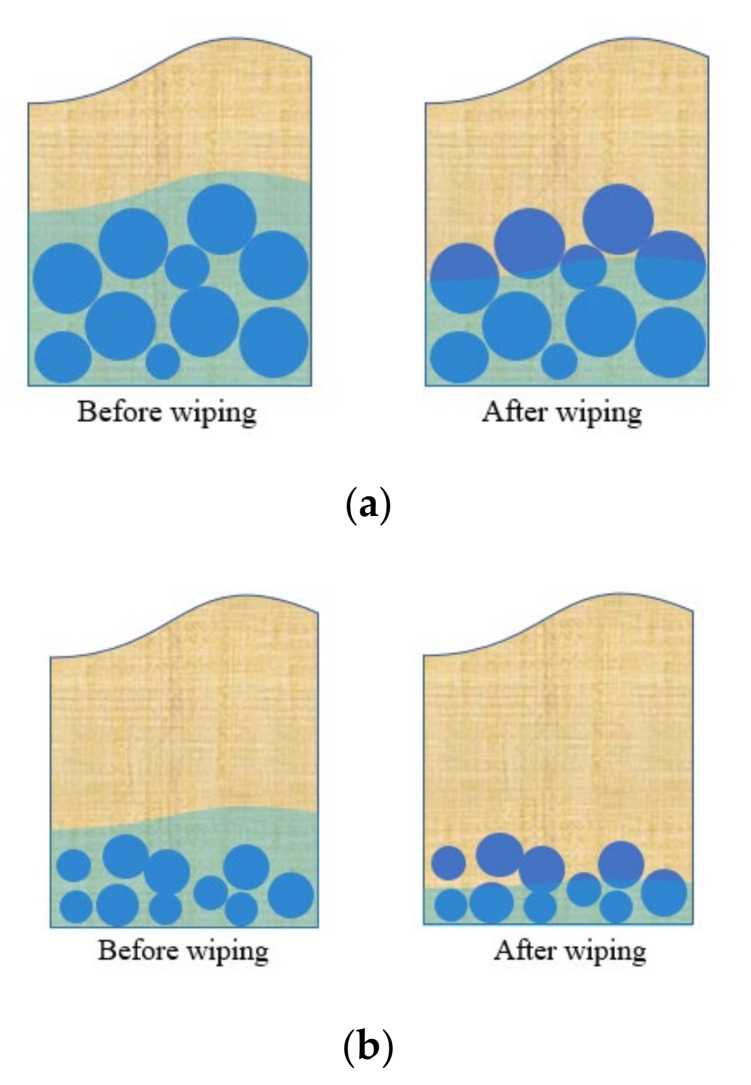
Inter-particle liquid removing process for the tea-bag method: (**a**) SAP A73 with a larger absorptivity and (**b**) SAP N with a lower absorptivity.

Interestingly, after 24 h contact with deionized water, the results of the tea-bag method were lower than the results of the filtration method (see Table 2). For SAP A73, the results of the tea-bag method were 1.4% lower when compared with the results of the filtration method. For SAP N, the results of the tea-bag method were 29.6% lower than the results of the filtration method. This indicated that the tea-bag method can remove more inter-particle liquid contrast to the filtration method. During the filtering process of the filtration method, most of the inter-particle liquid cannot be removed only by gravity. Zhao et al. studied the effects of both the filtration method and tea-bag method on removing inter-particle liquid with glass beads. The results exhibited that the amount of inter-particle liquid was higher when using the filtration method. For the tea-bag method, the inter-particle liquid is removed through wiping the surface of wet tea-bag for a short time [27]. As shown in Figure 4, the inter-particle space is larger for SAP A73 after being saturated due to the larger absorptivity. Thus, there is more inter-particle liquid within the sample of saturated SAP A73 particles. The short wiping time by a dry cloth can only remove a smaller proportion of the inter-particle liquid. The amount of inter-particle liquid that can be removed by a dry cloth is assumed to be the same. Then, the same wiping process can remove more proportion of the inter-particle liquid for SAP N due to its less inter-particle liquid. This may be the reason why the difference of absorptivity obtained by the tea-bag method and filtration method is more pronounced for SAP N.

**Figure 5:**
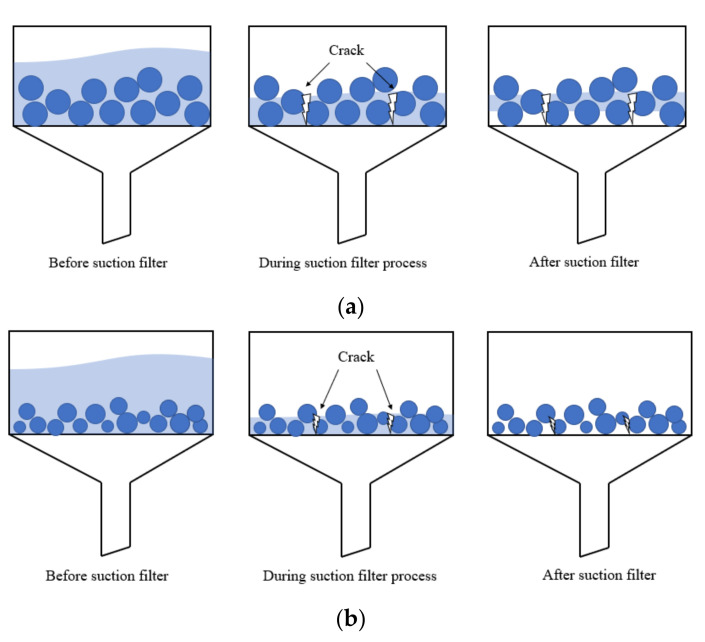
Inter-particle liquid removing process for the suction filtration method: (**a**) SAP A73 with larger absorptivity and (**b**) SAP N with lower absorptivity.

As expected, the 24-h absorptivity results in deionized water with both SAP types were systematically lower for the centrifuge method and the suction filtration method than for the tea-bag method and filtration method (see Table 2). For SAP A73, the centrifuge method and suction filtration method results were roughly 39.2% and 29.7% (approximately 156 g/g and 119 g/g) below those obtained from the tea-bag method. For SAP N, the centrifuge method and suction filtration results were roughly 41.6% and 36.9% (approximately 7 g/g and 6 g/g) below those obtained from the tea-bag method. Clearly, the centrifugal force of the centrifuge method and the suction pressure of the suction filtration method are more effective in removing the inter-particle liquid. Even though the reduction proportions for both SAP types were similarly high, the reduction values varied widely due to the different absorptivity. In other words, though the absorptivity results of SAP N from the tea-bag method is higher than the results obtained by the centrifuge method and suction filtration method, considering the scatter of results, the tea-bag method as a quick and simple testing method can still been used to predict the absorption kinetics of SAP N (low absorptivity). As shown in Figure 3a, for SAP A73, the absorptivity results obtained by the centrifuge method was still 13.6% lower than those obtained from the suction filtration method. However, this difference was not observed for SAP N. For the suction filtration method, the excess liquid (including free and inter-particle liquid) is removed by the suction pressure from the bottle of the funnel. As shown in Figure 5, SAP A73 will swell to a larger volume due to its higher absorptivity. When most of the excess liquid is removed from the funnel during the suction filter process, the cracks will form through the inter-particle space. Those cracks will intake air, which decrease the suction pressure. The lower suction pressure can only remove the inter-particle liquid close to the bottle of the funnel. Therefore, part of the inter-particle liquid still remains in the middle layer of the saturated SAP particles (see Figure 5a). However, this is not the case of SAP N due to its lower absorptivity (see Figure 5b).

### 3.2. Absorption of SAP in Cement Filtrate

**Figure 6:**
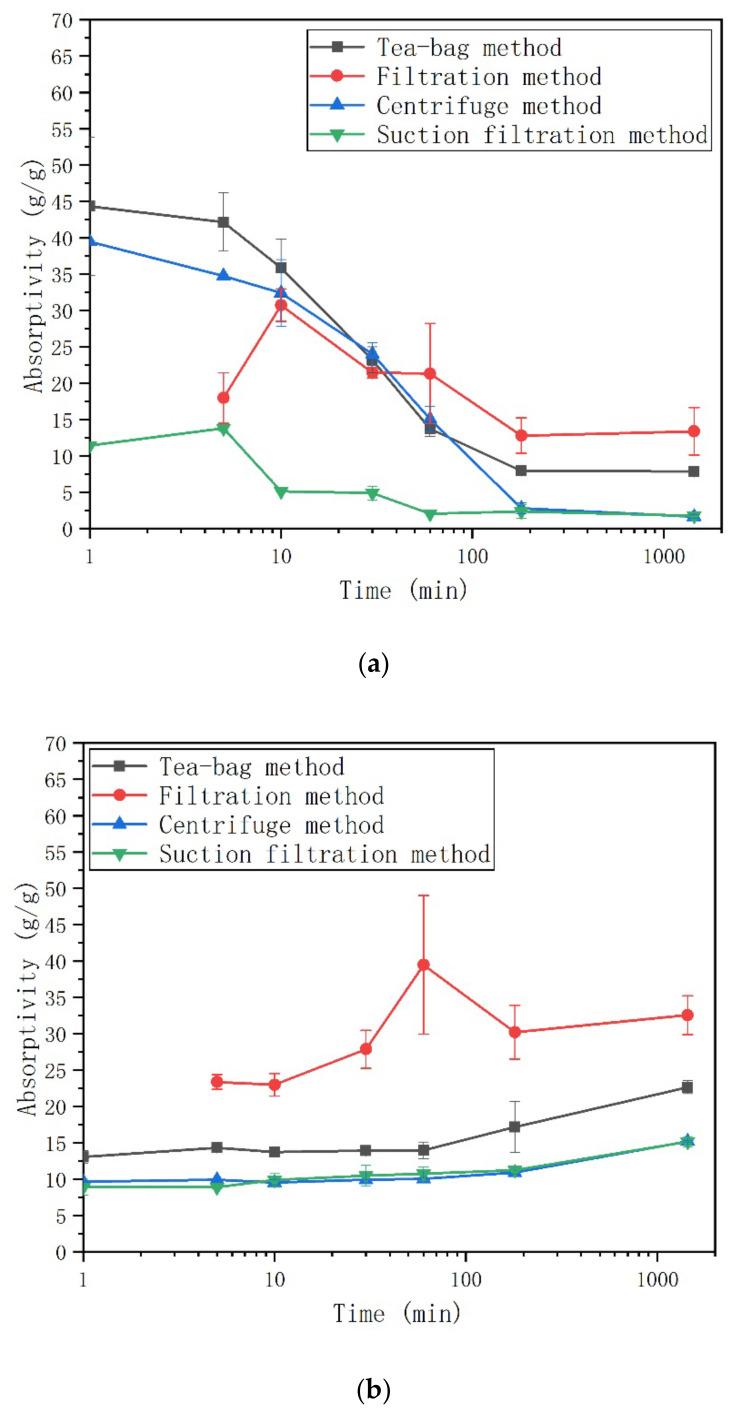
Development of SAP absorption in cement filtrate measured with different methods: (**a**) anionic SAP A73 and (**b**) AM-based SAP N.

As shown in Figure 6a, the absorption kinetics in cement filtrate for anionic SAP A73 exhibited rapid absorption behavior during the first minute. Then, a fast desorption behavior was observed. According to previous studies [22,45], this decrease in swelling behavior was not observed by the filtration method. However, in this study, the desorption behavior for SAP A73 can be clearly observed by all four methods (including the filtration method). This may be caused by the different liquid/SAP ratio used during the measurement [22]. A further reason may be a different SAP sample used in this paper. The acrylate groups on the network of SAP A73 will be complex with the cationic ion of Ca^2+^ in the cement filtrate. Then the cross-linking density of the network is increased [21,38,49]. This complexation will also decrease the efficient charge density of likewise anionic groups of the network of SAP [45,51]. For the same reason, the SAP N contain only amide groups, which will not complex with Ca^2+^ ions. Thus, the absorption kinetics of AM-based SAP N was relatively stable before 1 h and the absorptivity is even higher in the later age.

For SAP A73, the absorption results in the cement filtrate are much lower than that in deionized water. On one hand, the osmotic pressure between the internal and external of the network of SAP was reduced due to the dissolved ions in the cement filtrate [38,52]. On the other hand, the carboxylic group on the network of the polymer will complicate the Ca^2+^ ions in cement filtrate. The absorptivity will dramatically decrease [51]. For SAP N, only a minor difference was observed between the absorptivity in deionized water and in cement filtrate. Moreover, the absorptivity is even lower in deionized water when compared with cement filtrate during the later age. The amide groups on the chains of SAP N will be hydrolyzed to form carboxylic acid at a high pH (above 12) [53]. Thus, the driving force for swelling of SAP N in cement filtrate will be increased at a later age.

The same trend as in deionized water, higher scatter was observed in cement filtrate for the tea-bag method and filtration method when compared with the centrifuge method and the suction filtration method. For SAP A73, the scatter for the tea-bag method and filtration method ranges from 0.2 g/g to 10 g/g. The scatter for the centrifuge method and suction filtration method ranges from 0.1 g/g to 5 g/g. For SAP N, the scatter for the tea-bag method and filtration method ranges from 0.3 g/g to 9 g/g. The scatter for the centrifuge method and suction filtration method ranges from 0.1 g/g to 1.5 g/g. When the same method is applied, the scatter for SAP N is lower than that of SAP A73. In addition, when using the same batch of SAP A73 and an identical test method, higher absorptivity scatter was observed in deionized water when compared to that obtained in the cement filtrate. It seems that the absorptivity scatter is not only related to the method, but also related to the SAP types due to their different absorptivity.

The absorptivity results of SAP A73 obtained by the filtration method is lower when comparing the Tea-bag method and centrifuge method before 30 min. The final absorptivity at 24 h is higher than all other methods. Since the SAP samples are still exposed in the test liquid during the filtering process, the absorptivity measured at testing time may, in reality, be a later value. The absorptivity of SAP A73 in cement filtrate decreases over time. Hereafter, the absorption capacity decreased more due to longer contact time with cement filtrate during the early age. At a later stage, the delay of weighing due to filtering time have little influence on the absorptivity.

The absorptivity results of SAP A73 obtained by the suction filtration method is much lower than the other three methods (including the centrifuge method). In fact, part of wet SAP A73 particles adhere to the bottom and walls of the container with cement filtrate. These SAP particles cannot be completely washed into the Büchner funnel during measurement. Thus, the absorptivity results of SAP A73 were underestimated. It may be more accurate to evaluate absorptivity of SAP A73 by the mass of the liquid before and after water absorption (similar to the filtration method). However, a large amount of air is pumped into the Büchner flask during the suction filter process, which may also result in inaccurate absorptivity due to the serious carbonation of cement filtrate. Different from SAP A73, no clear adhesion particles of SAP N were observed on the bottom and walls of the container. Thus, the absorptivity results of SAP N obtained by the suction filtration method seems to give a truthful value.

The same trend as in deionized water, for both SAP types, the 24-h absorptivity results in the cement filtrate obtained by the filtration method were higher than by the tea-bag method (see Figure 6 and Table 3). This confirmed that a higher amount of inter-particle liquid was observed in the filtration method when compared with the tea-bag method. The same conclusions were observed from the tests in which deionized water were used for the test liquid. This is consistent with the results reported in the literature [22,45]. For SAP A73, the 24-h absorptivity obtained by the filtration method is 41.3% higher than by the tea-bag method. This difference is more pronounced when compared to that obtained in deionized water (see Figure 3a). As discussion in Section 3.1, a smaller proportion of the inter-particle liquid will remain within the inter-particle space of wet SAP particles due to its lower absorptivity (see Figure 4). The absorptivity of SAP A73 in cement filtrate is much lower than in deionized water. When the cement filtrate is used as a test liquid, more proportion of the inter-particle liquid of SAP A73 is removed with the same wiping process. For the same reason, though the 24 h absorptivity results of SAP A73 in cement filtrate were still higher for the tea-bag method than for the centrifuge method, the reduction values were not as much as in deionized water. For SAP N, the absorption capacity in cement filtrate obtained by the tea-bag method was roughly 30.5% below that obtained from the filtration method. This is consistent with the results in deionized water (approximately 29.6%), which can be explained by the similar absorptivity results between deionized water and cement filtrate for SAP N. The results indicate that the absorptivity results were not only influenced by the measuring method, but also influenced by the SAP types due to their different absorption capacity.

### 3.3. Absorption of SAP in Cement Paste

**Figure 7:**
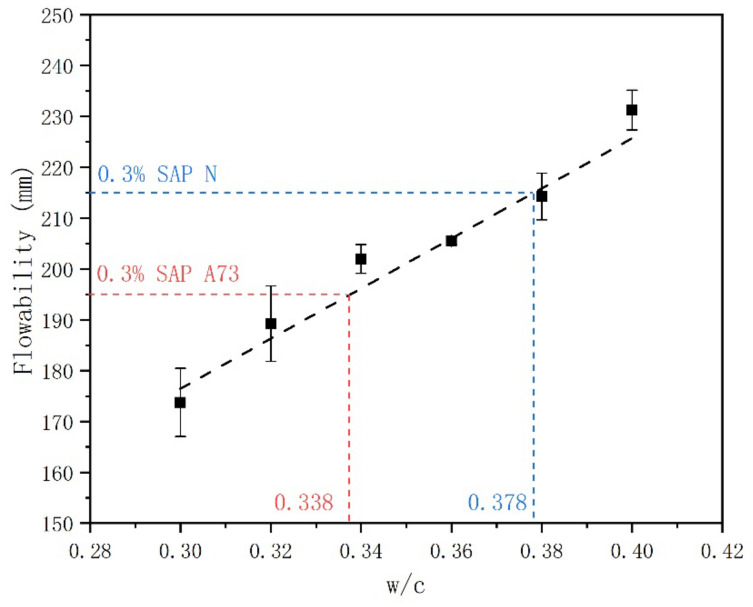
Relationship between w/c and flowability.

In Figure 7, the relationship between w/c and flowability is presented. As anticipated, the flowability of cement pastes increased clearly with the increase of w/c. Linear regressions were conducted based on the results of flowability, and the fitting line was also shown in Figure 7. The flowability of cement paste with SAP A73 and SAP N were 195 ± 1.4 mm and 215 ± 0.7 mm, respectively. Based on the fitting line, a similar slump flow for cement paste with SAP A73 and SAP N would be obtained when the w/c of cement pastes without SAP were 0.338 and 0.378, respectively. This results in an absorption of 20.78 ± 1.4 g/g for SAP A73 and 7.25 ± 0.7 g/g. When the four testing methods were compared with the slump method, it was found that the slump flow method yielded lower absorption values for both SAP types during the first 5 min of the absorption process. However, the centrifuge method and filtration method seem to be more accurate to predict the absorptivity of SAP in real cementitious materials. The swelling situation for SAP between in free liquid and realistic cement paste is different. In cement pastes, SAP was not exposed in infinite reservoirs of water anymore. In the cement paste with low w/c, as the supply of mixing water is limited, SAP will compete for the mixing water with cement particles during the process of water absorption. Therefore, the absorption and desorption kinetics in cement paste of SAP may be different with these in free liquid. The composition of pore solution is also influenced by w/c [54]. Therefore, the cement filtrate prepared at w/c = 5 is properly different with the pore solution in cement paste. According to the findings of Zhao et al. [22], there is no general trend between the slump and the dosage of SAP. However, a clear relation has also been found in previous studies [35,46] and this study. In addition, as the absorptivity of SAP A73 in cement filtrate is changed with time, the mixing time and casting time should also be considered when the slump flow method is used.

Considering the time consuming and experimental setups, both the tea-bag method and filtration method are more convenient to be used as preliminary approaches. However, for the tea-bag method, a possible error may occur since the inter-particle liquid cannot be totally removed by the wiping of dry cloth and the error can be even more pronounced when the SAP with higher absorptivity is used due to the larger inter-particle liquid. For the filtration method, since the SAP sample cannot be put back into the test solution after weighing, more of the SAP sample is needed to be consumed during the sorption test as a function of time. When compared with the tea-bag method, the filtration method was less appropriate for the absorption measurement. On one hand, the inter-particle liquid removed by gravity is less effective than by wiping by a dry cloth, which resulted in higher absorption results. On the other hand, the SAP samples are still immersed in the test liquid during the filtering process, so it is hard to estimate the accurate absorptivity at the early age. Though the centrifuge method and suction filtration method are more effective in removing the inter-particle liquid, they are harder to utilize and special setups are needed (i.e., centrifugal machine for the centrifuge method and vacuum pump for the suction filtration method). The centrifuge method seems to be more effective in removing inter-particle liquid when the SAP with high absorptivity is applied (see Figure 3a and Figure 5). For the slump flow method, it can predict the absorptivity of SAP in a real cement paste environment, but it is hard to estimate the absorption kinetics of SAP due to the limitation of mixing time and setting time. Especially for the anionic SAP (i.e., SAP A73), which will release the absorbed solution during the early time, the mixing time is a key factor. Thus, the accuracy of absorptivity results measured by the slump method need a further investigation. With this in mind, it is more reliable to estimate the absorptivity and desorption kinetic of SAP combined the centrifuge method and slump flow method. In addition, though the results obtained by the tea-bag method were less accurate, it is still a good way as a pre-test to reveal fundamental hints toward the absorption performance of SAP in the cementitious materials due to less time consumption and simple experimental setups needed.

## 4. Conclusions

In this paper, five approaches have been applied and compared to study the absorption capacity of two different types of SAP in different solutions, aiming for finding the optimum pre-test method to examine the performance of SAP as a concrete admixture.

The absorptivity results obtained from the tea-bag method and filtration method were found to be higher than that obtained by the centrifuge method and suction method. Furthermore, the scatter of the tea-bag method and filtration method were also higher when compared to that of the centrifuge method and suction method. The mechanisms behind these observations, which may be caused by the different ways to remove an inter-particle liquid. The centrifugal force of the centrifuge method and suction pressure of the suction filtration method seem to be more efficient to remove the inter-particle liquid. The SAP types with different absorptivity and the solutions also contribute to the results obtained by different methods.

The absorptivity results measured by the tea-bag method as well as the filtration method were highly influenced by the inter-particle liquid. In this study, the amount of inter-particle liquid in a modified tea-bag method (centrifuge method) is lower when compared with the tea-bag method. This difference is more pronounced when the absorptivity of SAP is lower.

In deionized water, the centrifuge method and suction method give more truthful absorption values for SAP N. However, the suction filtration method cannot remove all the inter-particle liquid for SAP A73 resulting in 13.6% higher absorptivity values compared to that of the centrifuge method. In cement filtrate, both the centrifuge method and suction method give more truthful absorption values for SAP N due to a similar low absorptivity being observed in deionized water. Due to the adhering issue of SAP A73 in cement filtrate, the suction filtration method underestimated the absorptivity. Thus, the centrifuge method seems to be a better testing method. It should be noted that the absorptivity in cement filtrate obtained by the centrifuge method and suction filtration were still higher than the absorption capacity in cement pastes obtained by the slump flow method due to the different absorption environments. In addition, slump flow cannot be used to monitor the absorption kinetics of SAP due to the limitation of mixing time. It is more reliable and recommended to estimate the absorptivity and absorption kinetic of SAP combined with the centrifuge method and slump flow method.

In addition, though the results obtained by the tea-bag method were less accurate, it is still a good way as a pre-test to disclose fundamental hints toward the effects and performance of SAP in the cement-based materials due to less time consuming and simple experimental setups needed.

## Figures and Tables

**Table 1:** Mixture proportion of the pastes.

Sample	w/c	SAP (by Mass of Cement)	PCE (by Mass of Cement)
R	0.3–0.4	-	0.15%
A73	0.4	0.3%	0.15%
N	0.4	0.3%	0.15%

**Table 2:** Final absorption at 24 h in deionized water.

Test Method	SAP A73 (g/g)	SAP N (g/g)
Tea-bag method	399.63 ± 22.19	16.61 ± 0.64
Filtration method	405.18 ± 8.13	23.60 ± 1.15
Centrifuge method	243.00 ± 4.15	9.70 ± 0.19
Suction filtration method	281.09 ± 4.92	10.48 ±0.12

**Table 3:** Maximum absorptivity and absorptivity at 24 h in cement filtrate.

Testing Methods	SAP A73 (g/g)		SAP N (g/g)
	Maximum	24 h	24 h
Tea-bag method	44.33 ± 9.49	7.85 ± 0.26	22.65 ± 0.98
Filtration method	30.73 ± 2.23	13.37 ± 3.28	32.57 ± 2.68
Centrifuge method	39.47 ± 0.13	1.65 ± 0.22	15.19 ± 0.08
Suction filtration method	13.78 ± 0.35	1.74 ± 0.42	15.16 ± 0.69
